# The influence of single-session reward-based attentional bias modification on attentional biases towards threat as measured by the N2pc component

**DOI:** 10.3389/fpsyg.2023.1279311

**Published:** 2023-11-20

**Authors:** Susan Kang, Roman Osinsky

**Affiliations:** Department of Psychology, School of Human Sciences, Osnabrück University, Osnabrück, Germany

**Keywords:** attentional bias modification, attentional bias for threat, N2pc component, EEG, single-session, reward-based, healthy participants

## Abstract

Attentional biases toward threatening faces have repeatedly been studied in the context of social anxiety, with etiological theories suggesting exacerbated biases as a possible cause for the latter. To counteract these postulated effects, research has focused on the concept of attentional bias manipulation (ABM), in which spatial contingencies between succeeding stimuli are traditionally employed in training paradigms designed to deliberately shift automatic attention processes away from threat-related stimuli. The ABM research field has been faced with various methodological challenges, such as inconsistent results, low reliabilities of dependent variables and a high susceptibility to moderating factors. We aimed to combine several recent approaches to address these issues. Drawing upon theories of value-driven attention, we explored reward-based contingencies in a Dot Probe task to improve the training’s efficacy, combined with neurophysiological measures for greater reliability compared to reaction times, while evaluating the moderating effect of explicitness in the instruction. In a healthy sample (*N* = 60) and within a single session, we found a general attentional bias toward angry faces present across all conditions as indicated by the N2pc, which was, however, marked by large intrinsic lateralization effects, with submeasures exhibiting opposing polarities. This prompted us to explore an alternative, intrahemispheric calculation method. The new N2pc variant showed the attentional bias to have disappeared at the end of the training session within the explicit instruction group. Reliabilities of the main dependent variables were varied from excellent to questionable, which, together with the exploratory nature of the analysis, leaves this result as preliminary.

## 1 Introduction

In a naturalistic environment, a multitude of stimuli of possible relevance are present at any moment. This entails the need for selective (visual) attention, a process that governs the distribution of limited cognitive resources, focusing them on the most relevant stimuli within the environment (Desimone and Duncan, 1995). The outcome of which particular stimuli succeed at capturing our attention is driven by various factors, broadly distinguished into top-down factors such as personal goals and context (Bacon and Egeth, 1994; Oliva et al., 2003), and bottom-up factors like a stimulus’ physical features and salience (Theeuwes, 1992; Itti, 2005). Here, we attempt to systematically manipulate the extent to which certain stimuli automatically capture participants’ attention by utilizing a related but separate construct, that of value-driven attention (Chelazzi et al., 2013; Bucker and Theeuwes, 2017). If successful, this procedure has potential implications for a field of research focusing on the etiology of social (in addition of other types of) anxiety disorder as well as its therapy.

Social anxiety disorder (SAD), also known as social phobia, is the most common anxiety disorder (Stein and Stein, 2008) with a lifetime prevalence of 4.0% across multiple countries in different geographical regions (Stein et al., 2017). Social anxiety disorder is marked by strong and persistent distress in social situations and fear of scrutiny by others, with respective situations being actively avoided. At the same time, the fear of (negative) social evaluation acts as a barrier to conventional treatments such as psychotherapy, and often prevents those afflicted from seeking treatment (Olfson et al., 2000).

It is therefore worth investigating alternative forms of intervention that do not rely on personal interaction and can be administered via computer-based training sessions, thereby lowering thresholds and making treatment more accessible and convenient. A possible such candidate is Attentional Bias Modification (ABM), a procedure based on the idea that emotional stimuli – such as threatening faces (Gamble and Rapee, 2010; Staugaard, 2010) – automatically capture attention (Bradley et al., 1997). These attentional biases are thought to be exacerbated in anxious individuals, and are postulated to play a role in the etiology and maintenance of (social) anxiety disorder (Rapee and Heimberg, 1997; Mathews and MacLeod, 2005; Van Bockstaele et al., 2014). ABM conventionally employs contingencies between target stimuli and differently valenced distractors to allow for measurement and manipulation of those biases, with the rationale that decreasing an inflated bias to normal levels should alleviate the symptomology of social anxiety disorder (MacLeod and Clarke, 2015). For measurement of attentional biases, the dot probe task (DPT) was developed by MacLeod et al. (1986), in which participants react to simple visual target stimuli replacing either threatening or neutral distractors with equal probability. Attentional bias is then operationalized as the difference in reaction times between the two conditions. For manipulation, a modified version of the DPT (MacLeod et al., 2002) is commonly used, which introduces spatial contingencies between distractors and targets such that targets appear at the location of neutral compared to negatively valenced distractors with higher probability.

While there has been a steady amount of interest in the field of ABM since its conception, it has been regarded more critically in recent years due to later studies often producing null results (e.g., Julian et al., 2012; Everaert et al., 2015), bringing its general effectiveness into question. In a meta-analysis, Heeren et al. (2015) found an overall small but significant effect of ABM methods on social anxiety symptoms, but criticized the quality of the studies as substandard, concluding that “ABM is not yet ready for wide-scale dissemination as a treatment for SAD in routine care.”

Multiple approaches to improve upon the procedure have been made since, two of which will mainly be focused upon here. Firstly, conventional measures of attentional bias (i.e., reaction time differences) have been criticized in terms of psychometric unreliability (e.g., Waechter et al., 2014; Waechter and Stolz, 2015). In an effort to identify reliable neurophysiological markers, a variety of event-related potentials have been investigated (Carlson, 2021). Among these, the N2pc component of the electroencephalogram (EEG), as first employed in an ABM approach by Osinsky et al. (2014), seems well suited due to its property of reflecting covert allocation of selective spatial attention between multiple stimuli (Eimer, 1996). Specifically, it has been shown to be elicited by task-irrelevant fearful faces (Eimer and Kiss, 2007), making it suitable for application in the dot probe task. The N2pc is typically observed as a transient negative deflection at occipitotemporal electrodes contralateral to the position of an attended stimulus 200 to 300 ms after stimulus onset (Luck, 2011). Its reliability to capture attentional biases in social anxiety was demonstrated by Reutter et al. (2017, 2019), indicating its potential usefulness as a measure in ABM training.

The other novel approach that will be focused on here is concerned with improving the efficacy of the attentional training task. Generally, it has been found that external (e.g., monetary) rewards can increase intrinsic motivation on low-interest tasks (Cameron et al., 2001), as which conventional ABM training has been frequently described by participants (Beard et al., 2012). More importantly though, rewards have been shown to impact visual selective attention, such that even task-unrelated stimuli increasingly capture attention after having been consistently associated with (higher) rewards (Libera and Chelazzi, 2006; Chelazzi et al., 2013). This effect may persist months after the initial training (Anderson and Yantis, 2013).

The underlying process, termed value-driven attention, is distinct from the top-down and bottom-up attentional systems mentioned previously and has the potential to act independently and even counteract these (Anderson, 2013; Bourgeois et al., 2017). It can thus be postulated that employing reward contingencies instead of the conventional spatial-probabilistic contingencies should (more) reliably achieve the desired effect of modifying attention to favor the higher rewarded stimuli in an ABM paradigm. The advantage of orienting attention toward the neutral distractor would therefore be the higher reward for correct reactions to a target following it, and not the higher possibility of the target appearing behind it. This idea has been tested in a first, albeit small, sample by Sigurjónsdóttir et al. (2015) who showed a reward-based training to be highly effective at manipulating attentional biases, while there was no effect of probability contingencies. In addition, and with regard to this method’s compatibility with the previously discussed approach, existing research demonstrates that these value-driven changes in attention can be captured by the N2pc component (Kiss et al., 2009). Changes in the N2pc’s amplitude have been shown to reflect preferential processing not only of simple stimuli associated with high rewards, but also that of complex objects (Donohue et al., 2016). It furthermore allows for tracking attentional adjustments caused by changing value-contingencies within a single experimental session (Oemisch et al., 2017).

Another moderating factor that has been studied in the context of ABM concerns the instruction given to the participants before performing the training task, in particular whether this instruction contains any explicit reference to the presence of a contingency between the stimuli. While the original ABM procedure did not inform participants of the contingency, as it was designed to address subconscious cognitive processes, some more recent studies have found an explicit instruction to be more effective in reducing attentional biases (*cf.* Krebs et al., 2010; Grafton et al., 2014; Nishiguchi et al., 2015). However, it has also been cautioned that explicit ABM, while more effective at lowering bias scores, might no longer have an impact on participants’ anxiety levels themselves (Grafton et al., 2014).

The present study aims to replicate and combine these novel approaches – improving upon both measurement and manipulation aspects of ABM – by measuring changes in the N2pc component in participants undergoing a reward-based attentional training. To maximize the generalizability of the results, we chose to study a healthy sample in a single training session (as opposed to preselecting for social anxiety or increasing the number of sessions). Lastly, as previous research has provided inconclusive results about the effects of informing participants about the presence of contingencies in the training condition, we also investigated the impact of implicit versus explicit instructions on the training’s efficacy.

## 2 Materials and methods

### 2.1 Participants

Sixty students (50 female; mean age = 21.9; SD = 2.4; one person’s demographic information missing) participated in the study and were reimbursed with course credit (where applicable) and monetary compensation, the latter of which was contingent on their individual performance in the task (around 15€). Power analysis performed in G*Power 3.1.9.7 (Faul et al., 2009) indicated that for the critical 3 × 3 mixed ANOVA using a significance criterion of α = 0.05, this sample size achieved a power of 0.99 when assuming a large effect size (Cohen’s *f* = 0.4) and a power of 0.86 when assuming a small to medium effect size (Cohen’s *f* = 0.2). According to self-reports, none of the participants had neurological or psychological conditions or were currently undergoing psychological treatment.

The study project was approved by the ethics committee of the University of Osnabrück and participants gave written informed consent.

### 2.2 Stimulus material and procedure

Stimulus presentation and behavioral data recording was controlled by PsychoPy (v2020.1.3) software (Peirce et al., **2019**). The task consisted of a dot probe paradigm (MacLeod et al., **1986**) that was modified to include (monetary) rewards for fast and correct responses. Barring this modification, stimuli and procedure were similar to those of Reutter et al. (**2017**). The stimulus material consisted of angry and neutral faces taken from the Karolinska Directed Emotional Faces database (Lundqvist et al., **1998**). Six female and six male models (AF01, AF09, AF19, AF20, AF22, AF26, AM02, AM05, AM10, AM11, AM14, AM29) were chosen and their respective angry and neutral expressions were used. A high perceptibility of the particular emotions on these specific models has been ascertained by Goeleven et al. (**2008**). Each trial started with a white fixation cross (whereas the background of the screen was gray) presented on its own for 500 ms. After this time period, two faces (“distractors”) were displayed horizontally on either side of the fixation cross (center at 3.72° visual angle, with a width of 4.75° and height of 6.41°). The two faces belonged to the same model but varied in their emotional valence with the three possible conditions being neutral/neutral, angry/neutral and neutral/angry (an angry/angry condition was not present). The distractors were displayed for another 500 ms, after which they disappeared and a colon (“target”) replaced either one of them with equal probability (50/50 chance of appearing in either the left or the right location). Participants were instructed to report via button press whether this colon was oriented vertically (:) or rotated by 90° (∙ ∙). Button assignments were counterbalanced across participants. The maximum response time started at 700 ms for each participant but was adapted according to individual performance, with a correct response lowering the limit by 50 ms and a wrong or late response extending it by 100 ms (up to a minimum/maximum of 400/1,200 ms). This adaptive response time limit was intended to enforce fast responses at the limits of participants’ reaction capacity. The target remained on screen for the full duration of the current response time limit or until a button press was made. Subsequently, a feedback was displayed in the middle of the screen (replacing the fixation cross). This feedback consisted of a phrase (“Correct,” “False” or “Too slow”) and, in brackets, the amount of money that had been or could have been received in this trial (e.g., “False (5 ct)”). In the case of a correct response, the text was colored green, otherwise it was white. The feedback was displayed for 1,000 ms, after which it disappeared and the screen was blank for an intertrial interval with a duration of between 750 and 1,250 ms (value drawn randomly from a uniform distribution).

**Figure 1:**
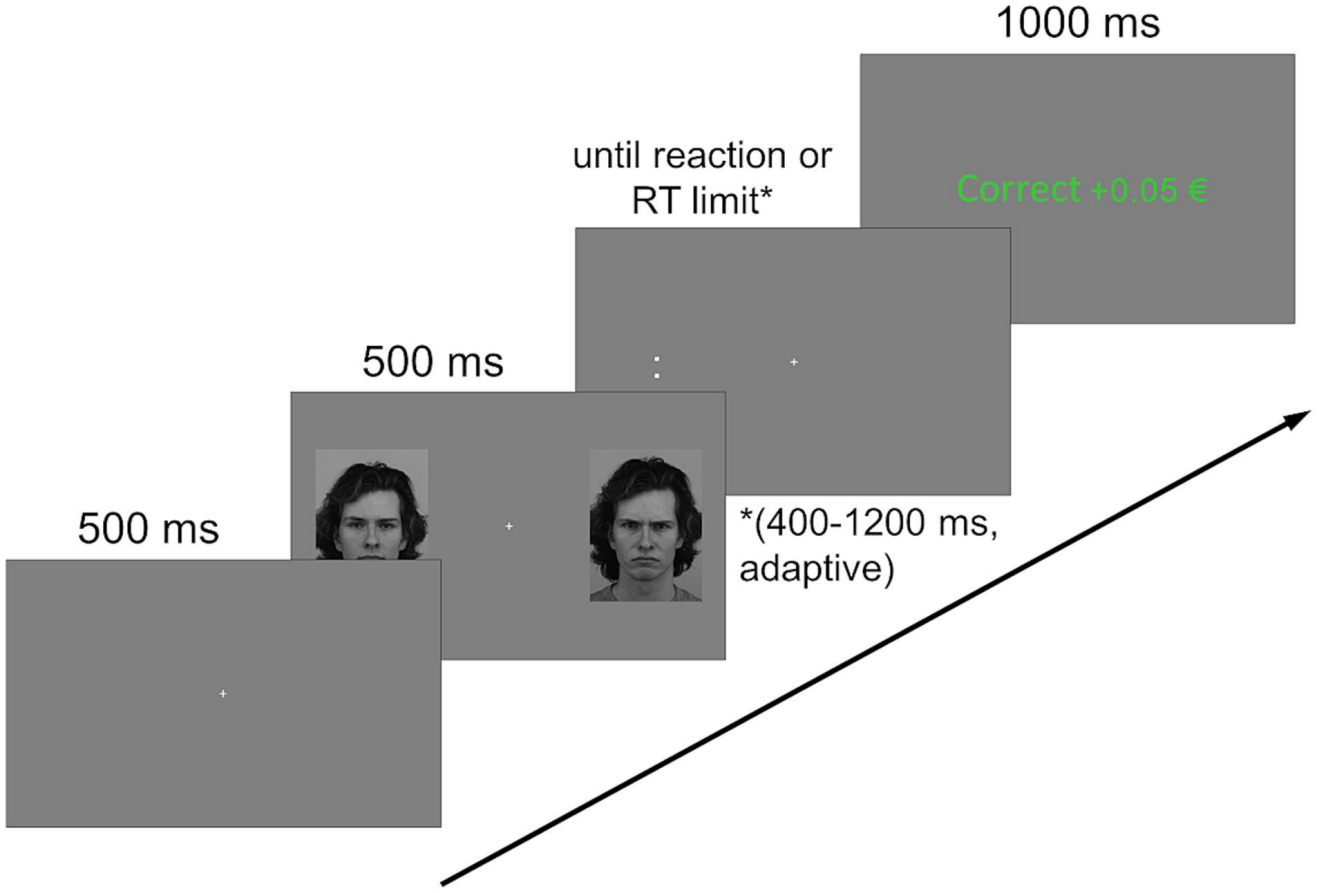
Exemplary trial sequence. A fixation cross, displayed for 500 ms, was followed by two distractors (faces of the same person with varying expressions) on either side of it, again displayed for 500 ms. A target stimulus replaced either of the distractors and stayed on screen until reaction or until an adaptive time limit (between 400 and 1,200 ms) was reached. Feedback depending on the (non-)reaction was then displayed for 1,000 ms, followed by an intertrial interval of 750 to 1,250 ms during which the screen was uniformly gray (not pictured) Facial stimuli shown here are image IDs AM14NES and AM14ANS, adapted with permission from the Karolinska Directed Emotional Faces database (Lundqvist et al., 1998).

In addition to model identities and distractor conditions, target location and orientation were counterbalanced across trials, resulting in 12 Models × 3 Distractor Conditions × 2 Target Locations × 2 Target Orientations = 144 trials per block. Each trial type (neutral/neutral, angry/neutral and neutral/angry) was presented an equal number of times, as such there were 48 trials of each type per block. An exemplary trial sequence is shown in Figure 1.

**Figure 2:**
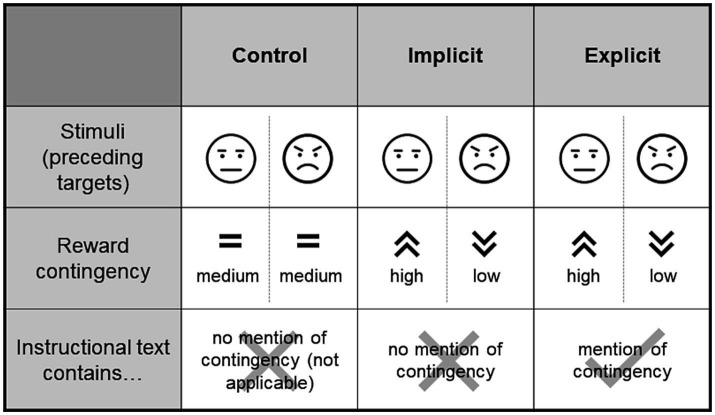
Experimental groups. The stimuli presented to each of the groups were identical, however, the groups differed with respect to whether or not there was a contingency in the amount of the reward that could possibly be collected when reacting quickly and correctly to the target following the stimulus (see Figure 1). Such a contingency (higher rewards for neutral faces and lower rewards for angry faces) was present for the training groups but not the control group. The training groups again differed within the instructional texts that were displayed prior to the task. In the “implicit” group, the contingency was not mentioned, however the “explicit” group made a reference to it. Schematic representations of angry and neutral stimuli are used in this figure, for an example of actual stimuli used see Figure 1.

Participants were assigned to one of three experimental groups (control, implicit, explicit). Those in the control group received a fixed reward of 5 ct for a correct response within the time limit. In the implicit group, the reward amount was contingent on the configuration of the distractor faces that preceded the target. If both faces were neutral, participants received a reward of 5 ct. If the face presented on the side of the target was angry (i.e., the opposing face was neutral), an amount of 2 ct was rewarded. In the converse case (i.e., a neutral face preceding the target with an angry face contralateral to the target) participants received 8 ct (given, in either case, that the reaction to the target was correct and sufficiently fast). These contingencies were designed to direct attention away from the negative faces toward the neutral ones by giving higher rewards in reaction to the latter, especially in cases where both expressions were present (i.e., participants had the choice of focusing on either). The explicit group received the same manipulation as the implicit group, with the only difference being an additional sentence in the instruction that alluded to the reward contingency but did not state it, with the aim of exploring whether this instruction (and consequently participants’ awareness of the contingency) affected the training’s effectiveness. Alluding to the presence of the contingency was intended to facilitate its subconscious perception and acquisition in accordance with the original idea of ABM, whereas an outright statement of the contingency might have rather engaged deliberate top-down control processes. The additional sentence translated to “The reward depends on the expression of the faces shown before.” Figure 2 summarizes the differences between the groups.

Prior to the experiment, participants completed questionnaires on their general demographic information, their trait social anxiety [Social Interaction Anxiety Scale/SIAS; Mattick and Clarke, 1998; German version by Stangier et al. (1999)] and reward sensitivity [Reinforcement Sensitivity Theory-Personality Questionnaire/RST-PQ; Corr and Cooper, 2016; German version by Pugnaghi et al. (2018); with only the items pertaining to the factor Reward Reactivity being included], in this order. The SIAS consists of 20 items such as “When mixing socially, I am uncomfortable.” that are rated on a 5-point scale ranging from “Not at all characteristic or true of me” to “Extremely characteristic or true of me” and has high internal consistency and retest reliability (Rabung et al., 2006). The RST-PQ is a 65-item questionnaire using a 4-point rating scale ranging from “Not at all” to “Highly” and has been demonstrated to be a reliable and valid measure (Pugnaghi et al., 2018). As mentioned before, from several scales included in the RST-PQ, only the Reward Reactivity scale was used here, which consists of ten items such as “I find myself reacting strongly to pleasurable things in life.”

Before the start of the recording, participants were allowed to practice the task in a training block that consisted of 10 random non-rewarded trials (using distractors that were not present in the actual experiment) until they performed at least 7 of these correctly. Following this, participants completed three blocks of the aforementioned 144 trials for a total of 432 trials, during which behavioral and EEG data were recorded. Trials were presented in random order within each block. There were self-paced breaks between and in the middle of all blocks. At the end of the experiment, participants filled out a post-test questionnaire containing some qualitative questions about the procedure.

### 2.3 Behavioral data processing

Erroneous trials (13.8%) and trials without reaction (18.6%) were excluded from the analysis for a total of 32.4% of removed trials. From the remaining correctly answered 67.6% of trials, outliers (reaction time exceeding 2 × *SD*) were removed separately for participants and conditions (i.e., group, block, distractor valence). Another 2.6% of the overall trials were removed in this step. The amount of remaining trials did not differ significantly between groups as shown by a one-way between-subjects ANOVA [*F*(2,57) = 1.38, *p* = 0.26, η_p_^2^ = 0.05].

The average reaction time for each cell was calculated from the remaining trials. These averages consisted of a minimum amount of 24 individual trials (mean = 31.23; max = 40). A bias score for angry faces was calculated separately for each participant and block by subtracting the average reaction time (RT) for neutral faces (i.e., trials, in which an angry and a neutral face were present and the target appeared behind the neutral face) from the average RT for angry faces (such that more negative values indicate a larger attentional bias for angry faces). This entails that the reaction times from the neutral/neutral conditions were not used in this calculation and are therefore not further included in the analysis of the behavioral data.

### 2.4 EEG recording and processing

The EEG was recorded using a 32-channel EEG system (Brain Products GmbH, Gilching, Germany) consisting of actiCap active electrode caps and a BrainAmp MR plus amplifier. The recording was performed using BrainVision Recorder v1.21.0303 software at a sampling rate of 500 Hz, with a band pass filter of 0.016 to 250 Hz and impedances that were kept below 20 kΩ. The 32 recording sites (Fp1, Fp2, F3, F4, F7, F8, Fz, FC1, FC2, FC5, FC6, C3, C4, Cz, CP1, CP2, CP5, CP6, P3, P4, P7, P8, Pz, TP9, TP10, T7, T8, PO9, PO10, O1, O2, Oz, according to the international 10–20 system) were referenced to FCz online, with the ground electrode placed at AFz.

Data were preprocessed offline using BrainVision Analyzer 2 (v2.2.1.8266) software. 0.1 Hz high-pass and 30 Hz low-pass Zero Phase Shift IIR Butterworth filters (24 dB/octave roll-off) were applied. For correction of ocular artifacts, an Ocular Correction ICA (extended biased infomax algorithm) was performed for all EEG channels. Components for which the sum of squared correlations with HEOG/VEOG exceeded 15% were excluded. HEOG and VEOG were operationalized as the difference between electrodes F7 and F8 and the mean of electrodes Fp1 and Fp2, respectively. Data were rereferenced to the average of all electrodes, with FCz being reinstated as an additional channel. For each block, data were segmented into epochs from-200 to 800 ms around distractor onset. Epochs that contained a voltage step of over 50 μV/ms were rejected, as were those with a maximum difference larger than 100 μV within 100 ms or those that reached an amplitude below or above −70/70 μV at any point. Similarly, epochs with a voltage difference exceeding 80 μV within 600 ms in the HEOG channel (to which the ICA had not been applied) were rejected to ensure that only trials without eye movements remained. Baseline correction was performed by subtracting the average signal in the time window from −200 to 0 ms before averaging the waveforms separately for each participant, block and distractor condition (i.e., neutral/neutral, angry/neutral and neutral/angry). These averages were calculated from a minimum of 20 trials each (of a maximum of 48 trials), with the mean being 43.99 (SD = 6.08). The case of a cell containing less than the 20 necessary trials occurred for less than 4% of cells overall, however, six participants were excluded from further analysis due to this. After the preprocessing, 19/19/16 participants remained in the control/implicit/explicit groups, respectively.

The N2pc component, which was chosen as the primary outcome measure due to its property of accurately reflecting selective spatial attention, was calculated in accordance with standards protocols. It was defined *a priori* as the difference in mean amplitudes at electrodes P7/P8 contralateral minus ipsilateral to the angry stimulus position 180–300 ms after distractor stimulus presentation. The choices of time and electrodes of interest were based on existing literature (*cf.* Eimer, 1996; Eimer and Kiss, 2007; Reutter et al., 2017). The N2pc was calculated in this way separately for each participant and block.

**Figure 3:**
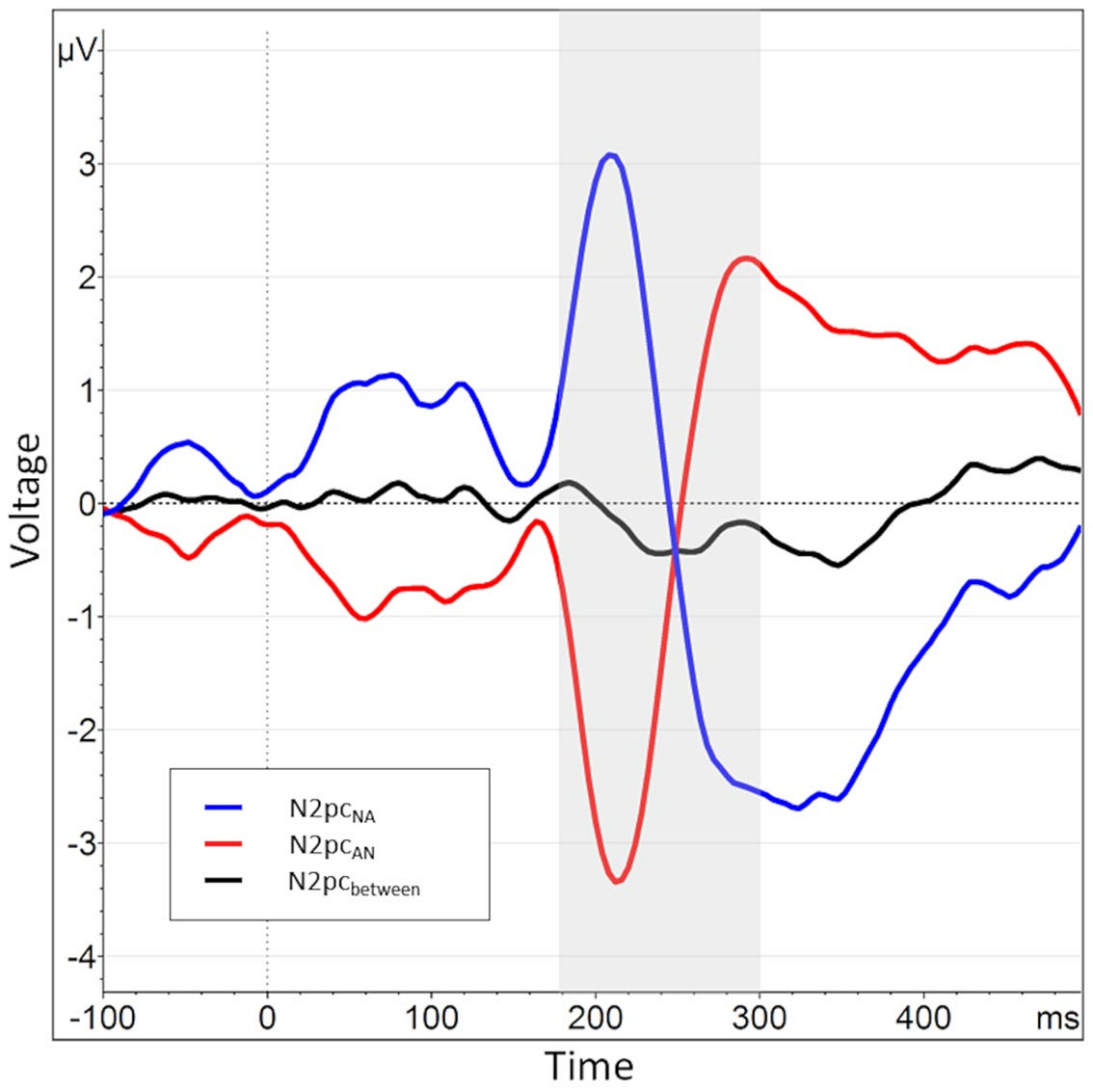
When splitting the N2pc_between_ into submeasures based on hemisphere, a pattern of near symmetry around the *x*-axis emerges, indicating a strong lateralization effect. The N2pc for angry faces presented in the right visual hemifield, i.e., processed in the left hemisphere, is shown in blue (P7_NA_ - P8_NA_). The N2pc for angry faces presented in the left visual hemifield, i.e., processed in the right hemisphere, is shown in red (P8_AN_ - P7_AN_). The N2pc_between_, the mean of these two submeasures, is shown again in black and manifests only as a small deviation from the symmetry.

In an additional, exploratory analysis, we also used a novel operationalization of the N2pc. This was motivated by a strong lateralization effect present in previous data collected in our group. In those data, angry faces presented in the left visual hemifield caused overall much larger deflections within their contralateral electrode (P8) than those presented in the right visual hemifield caused within P7. These lateralization effects were several times larger than the N2pc itself. This causes difference-based submeasures calculated from the two electrodes to be of opposite polarity depending on the order of subtraction (P8-P7 vs. P7-P8, *cf.* Figure 3).

To prevent this lateralization effect from masking actual experimental effects, we also calculated the N2pc not across hemispheres but within, utilizing the trials with neutral/neutral distractor configuration for that purpose. For instance, for the trials with angry/neutral distractor configuration, the mean amplitude at electrode P8 (i.e., contralateral to the angry face) was calculated as typical. However, the average amplitude at the same electrode (P8) during presentation of neutral/neutral distractors was subtracted from this, thus yielding the difference in activation between angry and neutral stimuli within the electrode contralateral to the angry distractor (note that accordingly, the ipsilateral stimulus was neutral in both cases).

This novel way of calculating the N2pc within single electrodes over different trials will subsequently be referred to by the label N2pc_within_, while the N2pc that was conventionally calculated as the difference between electrodes in a single trial will be called N2pc_between_.

To summarize the above, the two variants of the N2pc were calculated according to the following formulae, where the subscripts refer to the stimulus configurations of the trials included (A = angry, N = neutral):


$$N2pc_{\mathrm{between}}=\frac{N2pc_{NA}+N2pc_{\mathrm{AN}}}{2}$$

, where $N2pc_{NA}=P7_{NA}-P8_{NA}$ , i.e., electrode contralateral minus ipsilateral to angry stimulus for all trials with neutral/angry distractor configuration and. $N2pc_{\mathrm{AN}}=P8_{\mathrm{AN}}-P7_{\mathrm{AN}}$ , i.e., electrode contralateral minus ipsilateral to angry stimulus for all trials with angry/neutral distractor configuration. and $N2pc_{\mathrm{within}}=\frac{N2pc_{P7}+N2pc_{P8}}{2}$ , where $N2pc_{P7}=P7_{NA}-P7_{NN}$ , i.e., electrode P7 with an angry stimulus in the contralateral position minus electrode P7 with a neutral stimulus in the contralateral position and. $N2pc_{P8}=P8_{\mathrm{AN}}-P8_{NN}$ , i.e., electrode P8 with an angry stimulus in the contralateral position minus electrode P8 with a neutral stimulus in the contralateral position.

As described, all values indicated above refer to the mean activity at the respective electrode locations 180–300 ms after stimulus onset.

### 2.5 Statistical analysis

As indicated above, the main outcome measures used in our analyses were the N2pc (both variants), with reaction time bias toward angry faces being a secondary measure. The internal consistency of these measures was examined with a Monte Carlo-based split-half approach (Williams and Kaufmann, 2012) as performed by the *splithalfr* package (Pronk et al., 2022) with 5,000 repetitions, using data from the control group only (as per the study design, stability of effects over time could not be assumed for the other groups). Monte Carlo splitting involves the construction of two full-length data sets for each original data set based on random sampling with replacement (stratified by target category in our case), scoring the task and calculating the intercorrelation of the two series of scores across participants. This process is then repeated multiple times (i.e. 5,000 in our study) and the resulting Spearman-Brown corrected correlation coefficients averaged [see Parsons et al. (2019) for a discussion of the technique]. To assess the overall presence of attentional biases during the dot probe task, we performed one-sample, one-sided t-tests on all three measures, testing against zero. Mixed 3 × 3 factorial ANOVAs on all three measures with the between-subjects factor Group (control, implicit, explicit) and the within-subjects factor Block (1, 2, 3) were performed to assess group differences and potential changes in AB over the course of the experimental session. We also separately entered z-transformed SIAS and RST-PQ scores as covariates to account for the influence of the respective personality traits.

## 3 Results

### 3.1 Questionnaires

1.05% of questionnaire items were missing and thus interpolated by the mean of the other items. Sum scores in the SIAS questionnaire indicated a wide range of social anxiety levels from very low to high, with the majority showing low to moderate scores. Five participants had a score above 34 [cut-off value for social anxiety as suggested by Heimberg et al. (1992)]. Participants showed moderate to very high reward reactivity as specified by the RST-PQ sum scores. Descriptive statistics for both questionnaires are presented in Table 1.

**Table 1:** Descriptive statistics for the sum scores from SIAS and RST-PQ questionnaires.

Questionnaire	Mean	Min (Abs. Min)	Max (Abs. Max)	SD	Skewness	Kurtosis
SIAS	21.53	4 (0)	51 (80)	9.9	0.83	0.75
RST-PQ	29.72	22 (10)	38 (40)	3.91	0.16	−0.48

Missing items (1.05%) were interpolated by the mean of the respective participant’s other answers. Absolute min/max indicate the lowest/highest possible sum scores for the questionnaire.

### 3.2 Behavioral measures

The average winnings earned by participants amounted to 14.61€ (*SD* = 0.30€) and did not differ between experimental groups as indicated by a one-way between-subjects ANOVA [*F* (2, 57) = 0.44, *p* = 0.65, η_p_^2^ = 0.02]. The overall reaction time average for correct trials was 450.54 ms (*SD* = 66.49).

The average overall reaction time bias for angry faces was 0.6 ms (with a *SD* of 8.28 ms and a split-half reliability estimate of 0.72 (*SD* = 0.12) with a bootstrapped 95% confidence interval of [0.54, 0.86]). Lower values indicate speeded responses for angry faces, and thus the descriptive direction of the effect was in fact opposite to the predicted direction, albeit negligible (i.e., reactions to targets following angry faces were slower on average by less than one millisecond). This effect did not significantly differ from zero as shown by a one-sample two-sided t-test [*t* (59) = 0.56, *p* = 0.58, *d* = 0.07]. The mixed two-way ANOVA produced non-significant results, indicating that neither experimental condition [Group, *F* (2, 57) = 0.15, *p* = 0.86, η_p_^2^ = 0.02] nor time points [Block, *F* (2,114) = 0.10, *p* = 0.91, η_p_^2^ < 0.01] nor their interaction [*F* (4,114) = 0.56, *p* = 0.69, η_p_^2^ = 0.02] affected RT bias scores. Adding the SIAS and RST-PQ scores as covariates did not reveal a significant influence of these variables (all “*p*”s > 0.06).

### 3.3 N2pc

**Figure 4:**
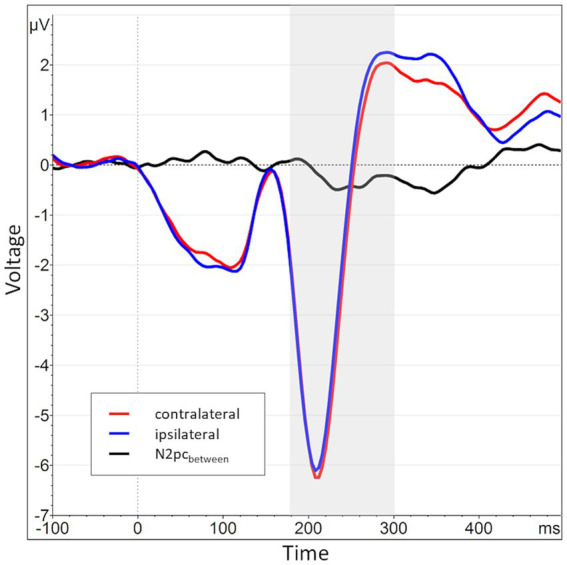
Grand average waveform of the N2pc_between_ waveform and its constituents across all participants and blocks at electrode positions P7 and P8. The time window of interest (180 to 300 ms post stimulus onset) is marked in gray. The average activity contralateral to angry face stimuli (e.g., at P8 for angry/neutral distractor configuration) is shown in red (mean of P8_AN_, P7_NA_), while the corresponding ipsilateral activity is shown in blue (mean of P8_NA_, P7_AN_). The N2pc_between_ (black) is the difference between contra- and ipsilateral activations.

**Figure 5:**
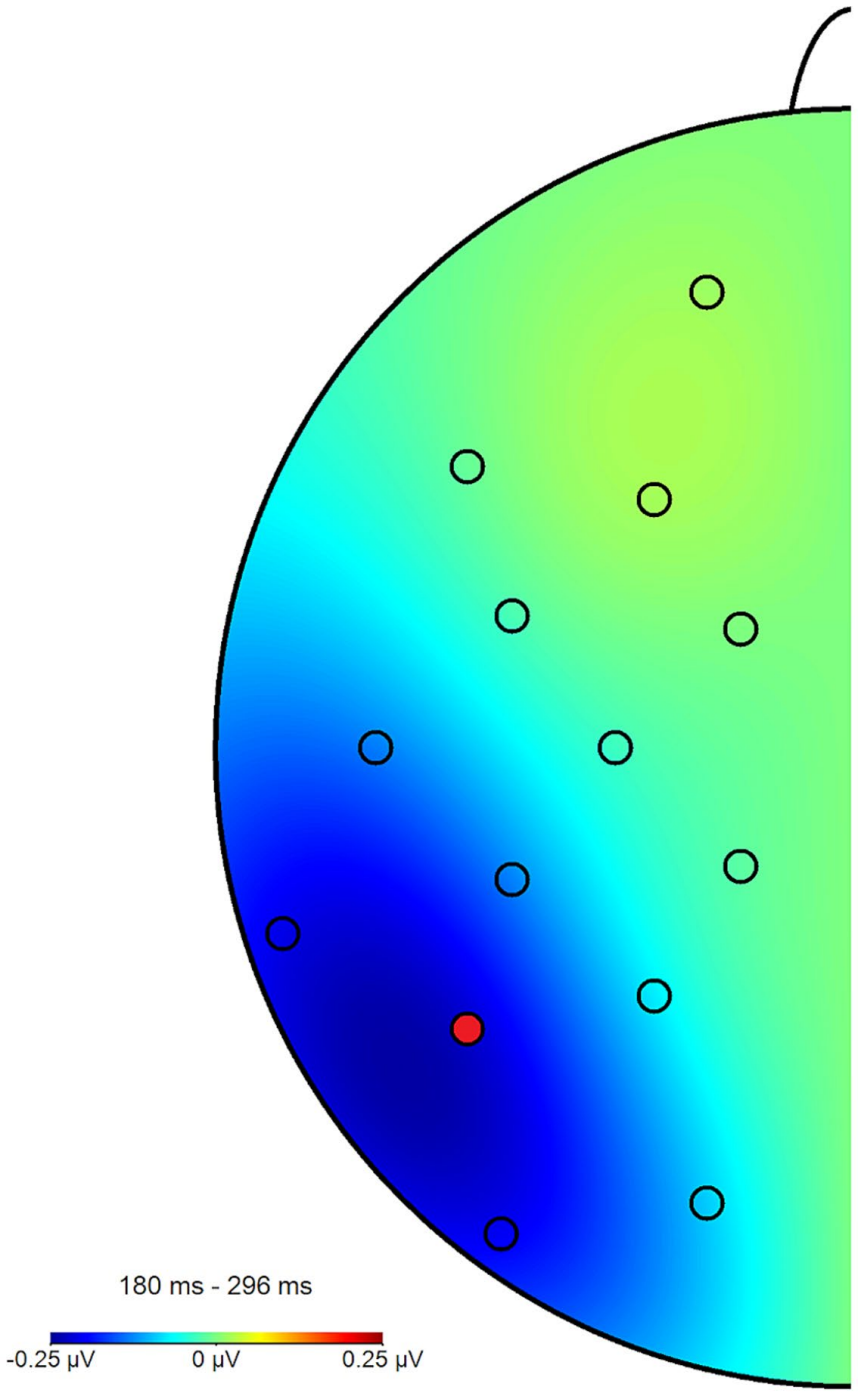
Topography plot of the N2pc_between_ effect across all participants and blocks. The electrode of interest (P7) is marked in red (P8 not pictured). For the purpose of this figure, difference waves from the angry/neutral and neutral/angry stimulus configurations were collapsed and mapped to the left side of the skull, such as if all angry faces had been shown in the right hemifield, when this was in fact counterbalanced.

**Figure 6:**
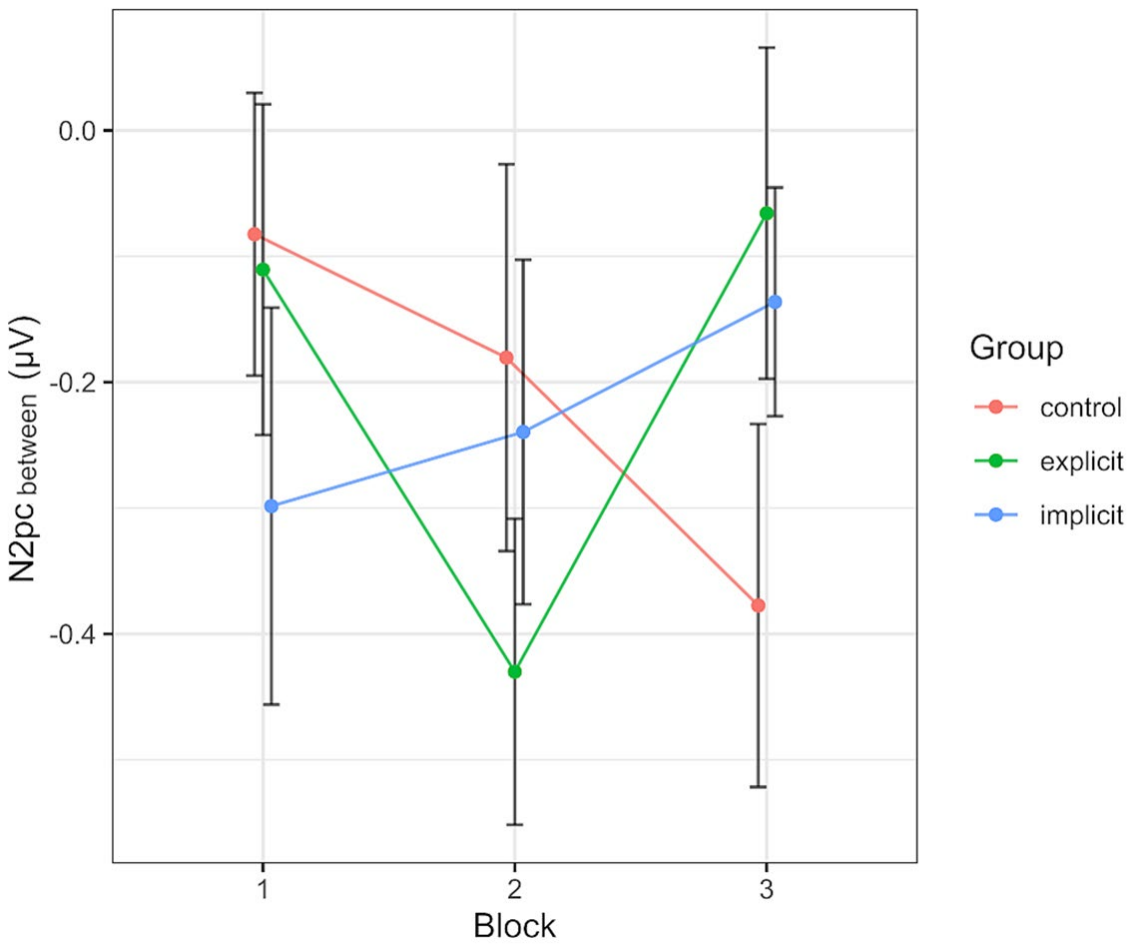
Progression of the N2pc_between_ over the three blocks, split by group. Each data point corresponds to the mean of the segment marked in gray in Figure 4 (regarding the black line). Error bars indicate SEM.

Across all participants and blocks, we found an average N2pc_between_ amplitude of −0.24 μV (*SD* = 0.37 μV), which indicated a generally stronger negative deflection in the hemisphere contralateral to the angry face stimulus compared to the ipsilateral hemisphere (see Figures 4, 5 shows the topology plot of this effect). This difference proved to be statistically significant as indicated by a one-sample one-sided *t*-test [*t* (59) = −4.9, *p* < 0.01, *d* = 0.64], suggesting the presence of a general attentional bias toward angry faces. Considering the average amplitudes separately by block and condition, the N2pc descriptively decreased in size (i.e., approached zero) over the course of the entire experiment in both the implicit and explicit training conditions. This decrease was continuous in the implicit condition, but not so in the explicit condition, where it is interrupted by a temporary shift into the opposite direction in block 2. In the control condition, the N2pc showed a steady descriptive increase in size (i.e., more negative values) with each block (see Figure 6). However, when performing the mixed two-way ANOVA with the factors Group and Block, the critical interaction of Group × Block did not reach significance [*F* (4,102) = 1.92, *p* = 0.11, η_p_^2^ = 0.07]. Neither of the two main effects were significant (Group [*F* (2,51) = 0.02, *p* = 0.99, η_p_^2^ < 0.01], Block [*F* (2,102) = 0.78, *p* = 0.46, η_p_^2^ = 0.02]). Adding the SIAS and RST-PQ into the model again did not yield significant results (all ‘*p*’s > 0.07).

The split-half reliability of the N2pc_between_ was estimated to be 0.82 (*SD* = 0.08, 95% CI [0.71, 0.89]). However, when considering the hemispheres separately (i.e., calculating the N2pc separately for trials with a left-sided vs. a right-sided angry face), these sub-measures displayed Spearman-Brown corrected reliability estimates of 0.98 (*SD* = 0.01, 95% CI [0.97, 0.99]) and 0.98 (*SD* = 0.01, 95% CI [0.96, 0.99]), respectively.

Regarding these sub-measures, we found the same hemispheric lateralization effect known from previous data (see section 2.4). That is, when plotted together, the two waveforms almost perfectly mirrored each other on the *x*-axis, with the collapsed N2pc merely representing a comparably minor deviation from this pattern (see Figure 3). This is reflected in a significant negative correlation of the two sub-measures, *r* (160) = −0.92, *p* < 0.01. Therefore, it seems that the side of data recording has *per se* a considerably larger influence on the variable of interest than the actual manipulation of stimulus position, possibly masking experimental effects.

**Figure 7:**
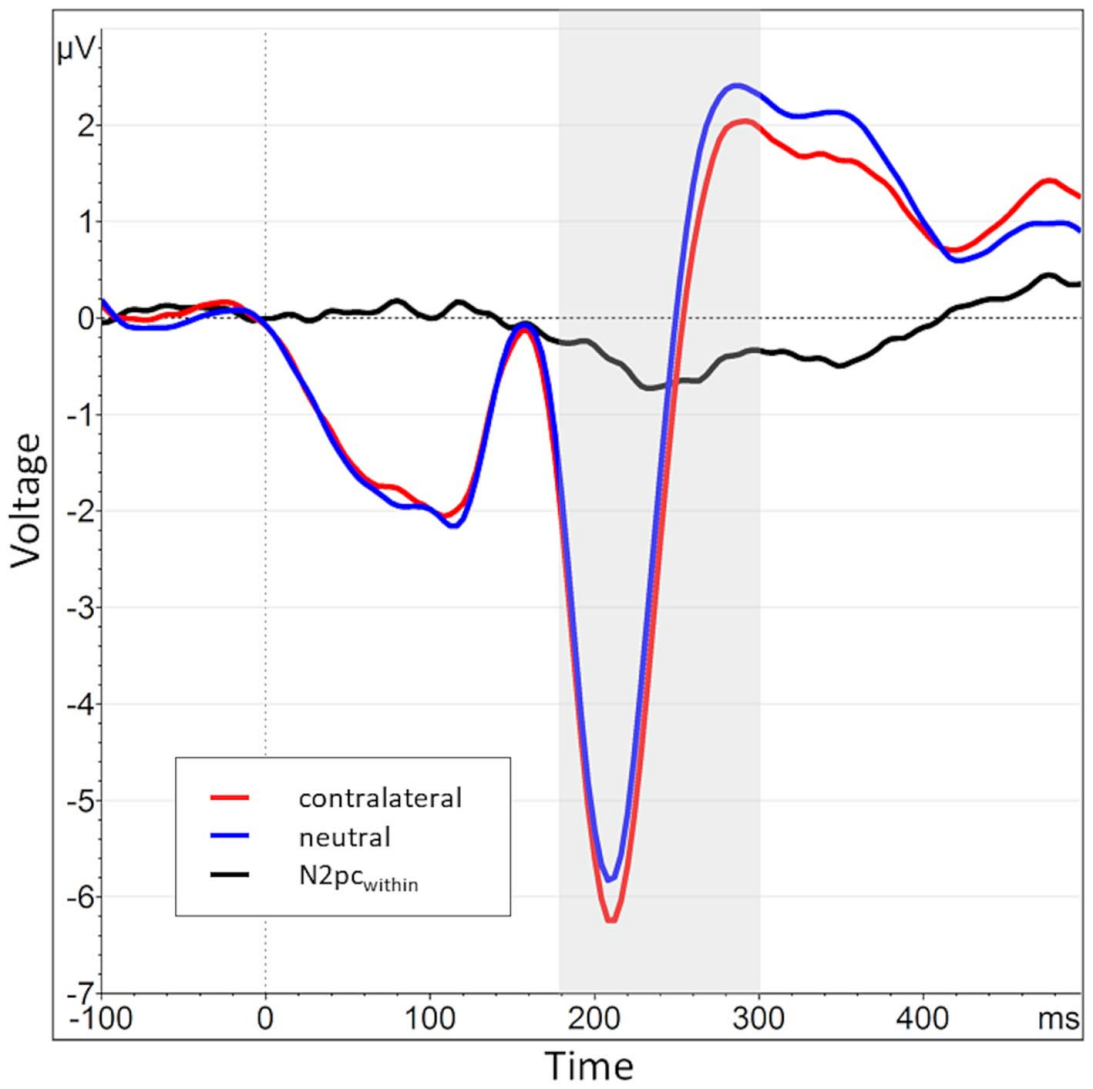
Grand average waveform of the N2pc_within_ waveform and its constituents across all participants and blocks at electrode positions P7 and P8. The time window of interest (180 to 300 ms post stimulus onset) is marked in gray. The average activity contralateral to angry face stimuli is shown in red (mean of P8_AN_, P7_NA_, identical to the red line from Figure 4), while the corresponding activity when both faces (i.e., also the contralateral one) were neutral is shown in blue (mean of P8_NN_, P7_NN_). The N2pc_within_ (black) is the difference between contralateral and neutral activations.

**Figure 8:**
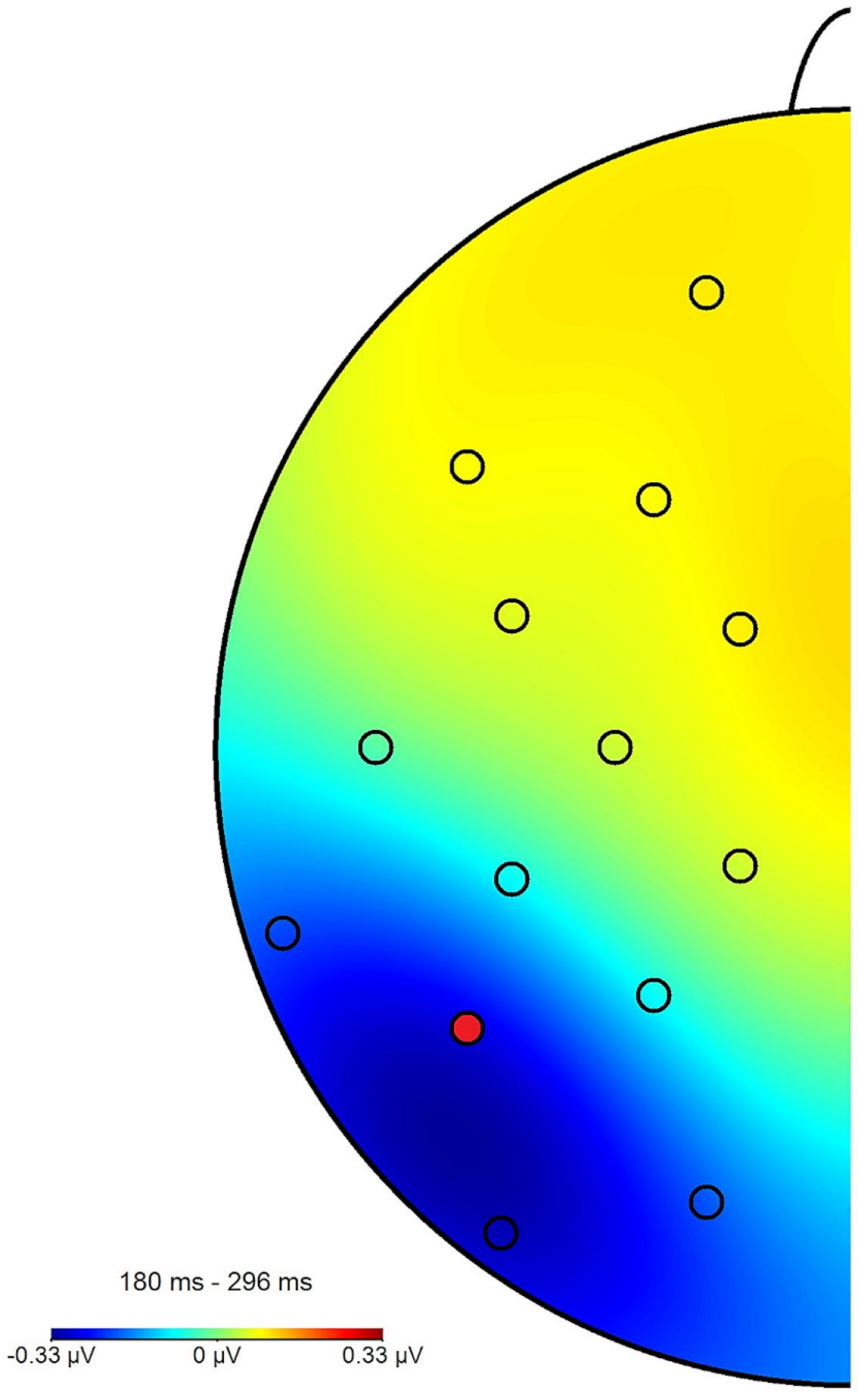
Topography plot of the N2pc_within_ effect across all participants and blocks. The electrode of interest (P7) is marked in red (P8 not pictured). For the purpose of this figure, difference waves from the angry/neutral and neutral/angry stimulus configurations were collapsed and mapped to the left side of the skull, such as if all angry faces had been shown in the right hemifield, when this was in fact counterbalanced.

**Figure 9:**
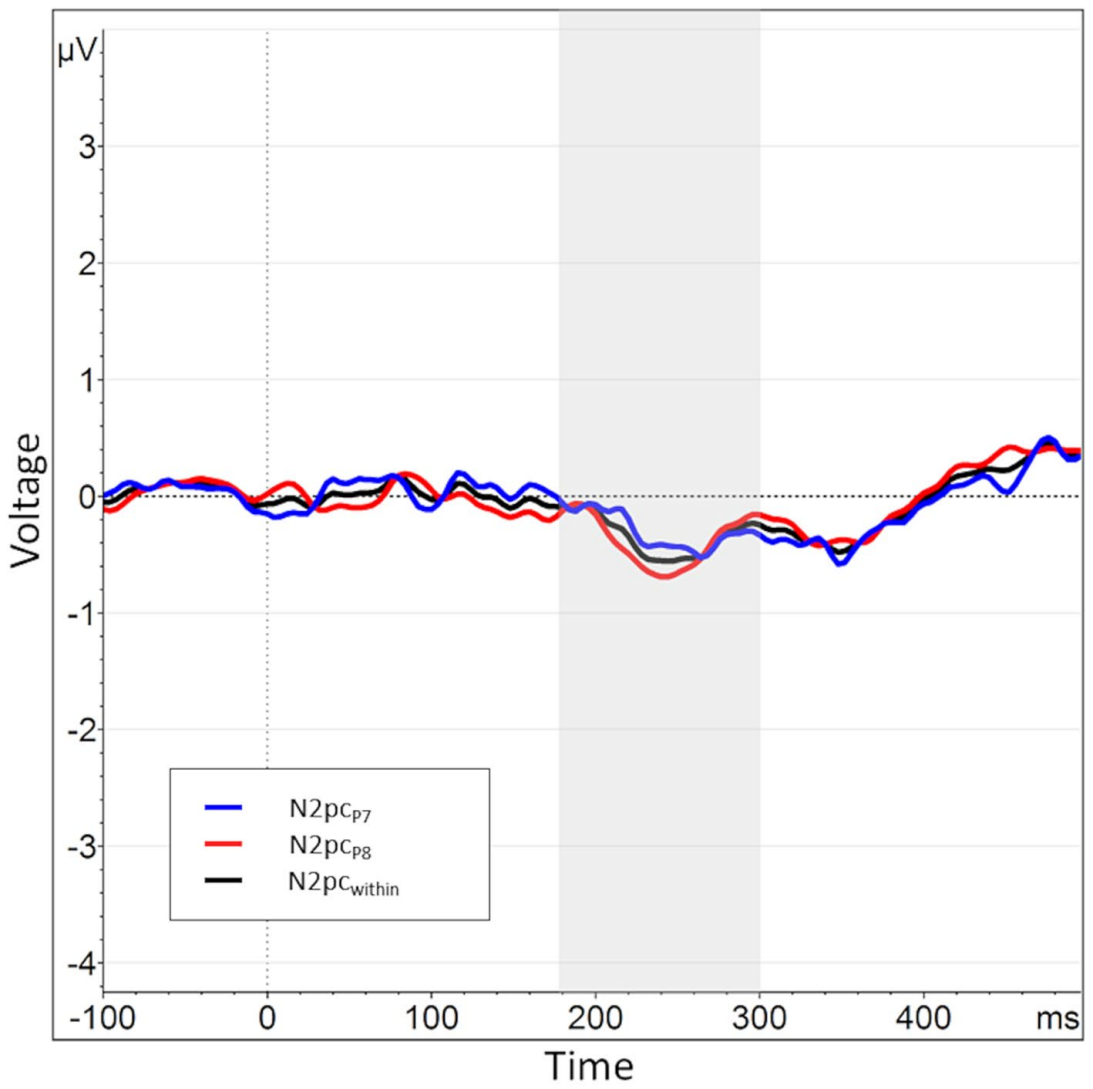
When splitting the N2pc_within_ into submeasures based on hemisphere, the submeasures show a high degree of similarity (as opposed to the submeasures of the N2pc_between_). The N2pc based completely in the left hemisphere, i.e., the difference in activation when an angry vs. a neutral face is shown in the right visual hemifield, is shown in blue (P7_NA_ – P7_NN_). The N2pc for the right hemisphere is shown in red (P8_AN_ – P8_NN_). The N2pc_within_, the mean of these two submeasures, is shown again in black.

The N2pc_within_ that was calculated to circumvent this lateralization effect (see section 2.4) had an average amplitude of −0.32 μV (*SD* = 0.4) across all blocks and conditions (see Figures 7, 8 for the topography). This difference was again shown to significantly differ from zero by a one-sample one-sided *t*-test [*t* (55) = −6.06, *p* < 0.01, *d* = 0.81], again reflecting the presence of a general attentional bias toward angry faces. The split-half reliability of the N2pc_within_ was estimated to be 0.64 (*SD* = 0.17), with a 95% CI of [0.52, 0.74]. Comparing submeasures of the N2pc_within_ based on hemisphere to each other (see Figure 9) showed that, as intended, these were not affected by the lateralization effect found for the N2pc_between_, with a significant positive correlation between the submeasures of *r* (160) = 0.21, *p* < 0.01. The reliability estimates for the submeasures were 0.7 (*SD* = 0.16, 95% CI [0.44, 0.84]) and 0.68 (*SD* = 0.13, 95% CI [0.56, 0.81]) for the left and right hemisphere (P7 and P8), respectively.

**Figure 10:**
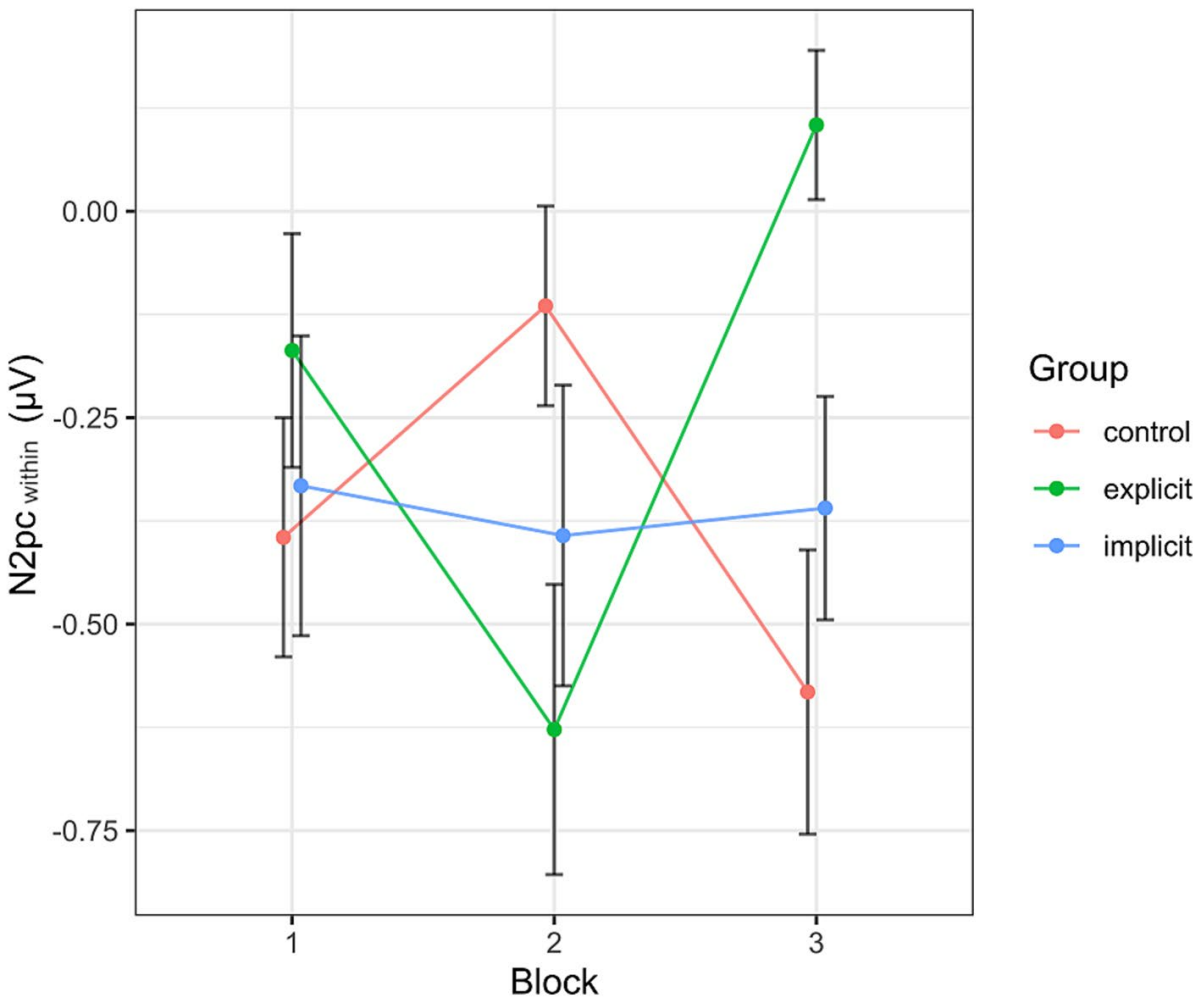
Progression of the N2pc_within_ over the three blocks, split by group. Each data point corresponds to the mean of the segment marked in gray in Figure 7 (regarding the black line). Error bars indicate SEM.

Inspecting the average amplitudes separately by block and condition, the revealed pattern (see Figure 10) differed from that described above for the N2pc_between_. Again, the N2pc descriptively increased in size in block 2 in the explicit condition, but was diminished in block 3, even reaching a positive value (indicating attention away from negative faces). The control group showed an inverted progression, decreasing in block 2 but again ending up at a more negative value in block 3 than at the beginning of the training session. The values in the implicit group stayed more or less the same across all three blocks. When performing the mixed two-way ANOVA with the factors Group and Block, there was a significant interaction of Group × Block [*F* (4, 102) = 4.07, *p* < 0.01, η_p_^2^ = 0.14]. The two main effects again did not reach significance (Group [*F* (2, 51) = 0.59, *p* = 0.56, η_p_^2^ = 0.02], Block [*F* (2,102) = 0.38, *p* = 0.69, η_p_^2^ = 0.01]. Adding the SIAS and RST-PQ into the model did not change the previous results and there was no significant interaction or main effect of either questionnaire (all “*p*”s > 0.14). Following up on the significant interaction by performing separate one-way ANOVAs with the within-subject factor Block for each group, the main effect Block reached significance in the explicit group [*F* (2, 30) = 6.08, *p* = 0.02, η_p_^2^ = 0.28], but not so in the implicit group [*F* (2, 36) = 0.05, *p* = 0.96, η_p_^2^ < 0.01] or the control group [*F* (2, 36) = 2.46, *p* = 0.1, η_p_^2^ = 0.12]. *Post hoc* pairwise comparisons between group means using Bonferroni correction showed a significant difference (reduction) in N2pc_within_ amplitude between blocks 2 and 3 within the explicit group (*p* < 0.01).

## 4 Discussion

We evaluated the effectiveness of reward-based attentional bias modification within a single training session using electrophysiological measures. Measuring the conventional N2pc as an index of selective attention toward angry face stimuli, we were able to identify a general attentional bias over all groups and blocks. However, for this conventional N2pc quantification we did not observe any substantive effects of the ABM training. Furthermore, the electrocortical activation seemed to be strongly characterized by lateralization effects as has previously also been reported by Reutter et al. (2019). This prompted us to explore an alternative calculation of the N2pc, combining values from within hemispheres rather than across. Over the course of the training session, we found a significant reduction of this measure during the second half of the training period, however only within the group whose instruction contained an explicit reference to the contingency between distractor valence and the level of reward associated with it.

Some of our findings are consistent with previous results. Specifically, the overall presence of an attentional bias toward angry faces as marked by (both variants of) the N2pc corroborates earlier studies (Eimer and Kiss, 2007; Feldmann-Wüstefeld et al., 2011; Kappenman et al., 2014; Osinsky et al., 2014; Reutter et al., 2017). Similarly, the absence of an attentional bias within reaction time data as well as their mediocre reliability has been shown repeatedly in recent research (Schmukle, 2005; Staugaard, 2009; Kappenman et al., 2014; Rodebaugh et al., 2016; Kruijt et al., 2019). It is worth noting that unlike the study sample in Reutter et al. (2017, 2019) and consistent with Osinsky et al. (2014), our sample was not preselected for high social anxiety scores, reinforcing the view that a certain level of attentional bias toward threat is found in the general population (e.g., Öhman et al., 2001; Schupp et al., 2004), not only within clinical or subclinical samples.

However, the main findings of this study raise several discussion points. Firstly, we must acknowledge that our manipulation did not have a significant impact on our main dependent variable, the conventional N2pc, but only on its exploratory variant instead. As previously discussed, this alternative quantification of the N2pc was calculated within hemispheres to increase sensitivity to possible experimental effects. To the best of our knowledge, there is no pre-existing literature examining this calculation method. Thus, it remains conjecture if both variants represent the same underlying neurocognitive mechanisms and can be interpreted equivalently. The basic logic behind both is consistent: a difference value based on posterior contralateral electrodes, with the minuend being the electrode involved in the processing of an angry stimulus and the subtrahend being the homolog (N2pc_between_) or identical (N2pc_within_) electrode during the processing of a neutral stimulus. However, it should be noted that for the conventional calculation method, every trial that entered the calculation contained both an angry and a neutral stimulus, whereas for the alternative method, the trials that constituted the subtrahend contained neutral stimuli only. Thus, the two methods differ on the very basis of the physical conditions involved. This may entail an interesting advantage of the exploratory method, in that, contrary to the conventional method, it allows for a comparison between the activity caused by an angry stimulus and a truly neutral condition.

Taken together, we believe that these considerations form the basis for interpreting the similarities and differences in results between the two variants: On the merit of following the same principle, the exploratory variant may be assumed to indicate selective spatial attention in the same way as the conventional N2pc does; as such both were able to showcase the general attentional bias present across all participants and blocks. Assuming the training effect found for the exploratory N2pc to be genuine, the most obvious explanation for why this did not manifest for the conventional variant (although exhibiting the same tendency) is the influence of the lateralized processes that prompted the alternative calculation in the first place. It is of note that hemispheric asymmetries have previously been reported in the context of the N2pc, having been speculated to reflect language-related lateralized processes due to the use of stimulus material with linguistic components (Eimer, 1996; Liu et al., 2009). Accordingly, in the present study, the lateralization effects might have been a consequence of the right-hemispheric dominance known for facial processing (Hay, 1981; Kanwisher et al., 1997; Prete and Tommasi, 2018).

Taking a closer look at the pattern of results obtained for the exploratory N2pc_within_, its disappearance at the end of the training session in the explicit group indicates that the reward-based training was effective insofar as that threatening faces were no longer processed preferentially at this point. The fact that we found this significant reduction only within the explicit training condition might be taken to indicate that conscious knowledge of the reward contingencies is necessary to achieve the desired training effect. Yet, in the post-test questionnaire, the vast majority of participants in the explicit as well as the implicit condition stated not having been aware of any contingency during the experiment (there were only two to three exceptions in either group). Thus, it cannot be concluded that the reason for the significant effect within the explicit group lies within explicit awareness of the training contingency. It might however be the case that the allusion toward the contingency primed participants in the explicit group and facilitated their subconscious perception of it. Indeed, in order to address attentional biases which are subconscious processes themselves, ABM has been designed to operate on the subconscious level in its original conception, with the use of implicit instruction being the standard especially in its early stages (see, e.g., Hakamata et al., 2010). On the other hand, this approach has been challenged by several studies who found an advantage for explicit training methods (Krebs et al., 2010; Nishiguchi et al., 2015). Note that regardless of the outcome of this debate in the traditional ABM literature, in the context of *reward-based* ABM, the question of which method is superior remains up for debate, as the same results do not necessarily hold for value-driven attention (it’s not unreasonable to assume that awareness of reward contingencies in this context might make them more effective). An alternative to the previously attempted explanation, i.e., facilitation of subconscious perception, for the differential outcome between implicit and explicit training groups might be that participants in the explicit training group were in fact consciously aware of the reward contingencies as a result of the instruction, yet failed to mention so in the post-test questionnaire for whatever reasons. In conclusion, while the present results do imply an advantage for an explicit over an implicit instruction, the uncertainty with respect to the participants’ actual levels of contingency awareness as well as the exploratory nature of the dependent variable prevent a conclusive explanation of this effect.

Another point that should be addressed are the reliability estimates associated with the various dependent variables. The low reliabilities of reaction time biases in previous research have generally drawn criticism and been a major reason for the attempt to switch to other modalities. In the present data set, this has been met with partial success. While the two subcomponents of the conventional N2pc displayed nearly perfect reliabilities by themselves, the collapsed measure scores lower, while still being in a viable range. The exploratory N2pc measures had even lower reliabilities, both the collapsed as well as the intrahemispheric submeasures, being of overall questionable reliability. Interestingly, the RT bias displayed higher reliabilities than the exploratory N2pc measures, in fact being overall acceptable. To interpret these patterns, it helps to take a look at the underlying properties of the reliability assessment. The fact that reliabilities are estimated by correlating (subsets of) a dataset with itself means that high scores are contingent on the presence of stable between-subjects variability. That is, even if a task itself produces robust effects, if these effects are too similar between participants, reliability estimates will be low. While low between-subjects variability is usually coveted, leading to stable and replicable effects, it paradoxically also means that it causes problems for the use in a correlational context (Hedge et al., 2018). Furthermore, the systematic and stable variance between participants that leads to high reliability scores can be introduced both by effects of interest and by other factors that are not relevant to the study question. Conversely, they are impacted by the introduction of unsystematic variance or noise. In the context of the present study, the lateralization effect caused by face processing is an example for a factor of no interest. Its presence, however, results in large and stable between-subjects variability. This explains why the conventional N2pc submeasures display such high reliabilities, as they include the lateralization effect which even gets exacerbated by the subtractions involved in the calculation. The collapsed conventional N2pc on the other hand displays lower reliability because the systematic variance introduced by the lateralization effect is canceled out. This however may actually be seen as an advantage, since the remaining reliability is more likely to truly reflect that of the effect of interest. Finally, the exploratory N2pc circumvents the influence of lateralization effects as it was intended to do, thereby losing systematic variance (of no interest). At the same time, and as mentioned above, it uses physically different trials, while for the conventional N2pc, the difference is taken between electrodes within the same trial, meaning that random inferences in the signal are largely subtracted away. The latter does not work for the calculation of differences across trials, thus introducing a higher level of random noise. These two considerations may explain why the exploratory N2pc has the overall lowest reliability. Taken together, the assumed pattern is as follows: submeasures of the conventional N2pc include high systematic variation of interest, high systematic variation of no interest and low noise and therefore display excellent reliability. The collapsed conventional N2pc includes high systematic variation of interest, low systematic variation of no interest and low noise and therefore exhibits good reliability. The exploratory N2pc and its submeasures include high systematic variation of interest, low systematic variation of no interest and high noise and thus show questionable reliability. As a final comparison between the two variants of the N2pc, the exploratory variant may be described as more sensitive but less reliable (but see the reliability paradox mentioned above).

The current study aimed to find evidence for a reward-based ABM training’s effectiveness under the simplest and most general conditions, that may have acted as limitations at the same time (a single training session, no preselection of participants and limited sample size), in the hope that if successful, these findings could be extrapolated to the other cases as well (with the inverse not necessarily being the case). In our case, due to the partially exploratory nature of the analyses as well as the inconclusive pattern of the results and reliability measures, the presented finding of a significant attentional bias reduction in the explicit training condition should be treated as preliminary. Further studies could take the inverse approach and maximize each of the parameters (i.e., a high number of training sessions in a large, preselected sample) to provide conclusive evidence for or against the potential efficacy of reward-based ABM under the most facilitative conditions.

## Data availability statement

The raw data supporting the conclusions of this article will be made available by the authors, without undue reservation.

## Ethics statement

The studies involving humans were approved by the Ethics Board of the University of Osnabrück. The studies were conducted in accordance with the local legislation and institutional requirements. The participants provided their written informed consent to participate in this study.

## Author contributions

SK: Formal analysis, Investigation, Project administration, Writing – original draft, Software. RO: Conceptualization, Funding acquisition, Supervision, Writing – review & editing.
